# Comparative genomic analysis of the *bla*_OXA-23_-carrying plasmids

**DOI:** 10.1128/spectrum.01374-26

**Published:** 2026-09-29

**Authors:** Jiahui Weng, Xiaobin Li, Ziyue Yang, Huiting Huang, Shaofeng Zhan

**Affiliations:** 1 The First Affiliated Hospital of Guangzhou University of Chinese Medicine 47879, Guangzhou, China; 2 The First Clinical Medical College, Guangzhou University of Chinese Medicine, Guangzhou, China; 3 Guangdong Provincial Key Laboratory of Tumor Interventional Diagnosis and Treatment, Zhuhai People’s Hospital (The Affiliated Hospital of Beijing Institute of Technology, Zhuhai Clinical Medical College of Jinan University) 198044, Zhuhai, China; 4 Department of Pulmonary and Critical Care Medicine, Zhuhai People’s Hospital (The Affiliated Hospital of Beijing Institute of Technology, Zhuhai Clinical Medical College of Jinan University) 198044, Zhuhai, China; Innovations Therapeutiques et Resistances, Toulouse, France

**Keywords:** plasmid-borne *bla*_OXA-23_, *Acinetobacter baumannii*, genetic context, comparative genomic analysis

## Abstract

The global dissemination of the carbapenem resistance gene *bla*_OXA-23_ is partly facilitated by plasmids, yet a comprehensive characterization of these vectors is lacking. This study analyzed the *bla*_OXA-23_-carrying plasmids selected from the National Center for Biotechnology Information RefSeq database to elucidate their general characteristics and genomic diversity. The *bla*_OXA-23_-carrying plasmids were predominantly hosted by *Acinetobacter baumannii* (94.06%), and their sizes varied widely, from 8.22 to 336.05 kb. Replicon typing revealed that the majority belonged to the RP Rep family, specifically RP-T1 (62 plasmids) and RP-T2 (23 plasmids) types. A key finding of this analysis was the assignment of these two major plasmid groups to distinct plasmid taxonomic units (PTUs): all RP-T1 plasmids were classified as PTU-Pse5, whereas all RP-T2 plasmids were classified as PTU-Pse1. The *bla*_OXA-23_ gene of the plasmids was located within four kinds of composite transposons, with Tn*2006* (33.67%), Tn*2008* (32.67%), and Tn*2009* (31.68%) being the predominant genetic contexts. Notably, the 62 plasmids of RP-T1 type (PTU-Pse5) and the 23 plasmids of RP-T2 type (PTU-Pse1), found predominantly in *A. baumannii*, carried genes encoding relaxases of the MOB_F_ family, T4CPs of t4cp2, and type F T4SS, suggesting they are putatively conjugative. In addition, the 62 plasmids of RP-T1 type (PTU-Pse5) were identified in multiple countries within the data set, while the 23 plasmids of RP-T2 type (PTU-Pse1) were predominantly found in China. These findings provide a genomic view of *bla*_OXA-23_ plasmids, suggest their role in disseminating carbapenem resistance across species and regions, and offer a surveillance framework, highlighting key lineages for clinical intervention.

**IMPORTANCE:**

The horizontal transfer of *bla*_OXA-23_ to *Acinetobacter baumannii* and other gram-negative pathogens has conferred high-level carbapenem resistance, posing significant therapeutic challenges in both hospital and community settings. Plasmids carrying conjugative transfer systems serve as key vehicles for the dissemination of *bla*_OXA-23_ among clinical isolates. In this study, we identified the plasmid lineages responsible for *bla*_OXA-23_ spread and categorized them into a limited number of groups. This finding underscores the considerable risk of horizontal gene transfer in propagating carbapenem resistance and provides a valuable framework for future surveillance efforts to track the transmission of these resistant plasmids. However, this study was based on current knowledge and available data. Given the ever-changing situation of antibiotic resistance, new plasmid variants or transfer mechanisms may emerge, potentially limiting the long‑term applicability and accuracy of our findings.

## INTRODUCTION

Carbapenem-resistant *Acinetobacter baumannii* (CRAB), which is a major human pathogen associated with high mortality rates (1), remains listed as a critical priority pathogen in the World Health Organization (WHO) priority pathogen list released in 2024 (2). The primary resistance mechanism for CRAB is the production of carbapenem-hydrolyzing class D β-lactamases (CHDLs), particularly OXA-23 (3).

First identified in an *A. baumannii* isolate in 1985 (4), OXA-23 has since been reported globally in both *A. baumannii* (5) and other *Acinetobacter* non-*baumannii* species (6). In addition, OXA-23 has also been reported in non-*Acinetobacter* species, such as *Proteus mirabilis* (7). The global dissemination of OXA-23 has substantially contributed to the burden of carbapenem resistance (1, 8). The *bla*_OXA-23_ gene has been found on a variety of composite transposons, mainly Tn*2006*, Tn*2007*, Tn*2008*, and Tn*2009* (9). These transposons share a common region harboring the *bla*_OXA-23_ and an ATPase gene. While Tn*2007* contains IS*Aba4* upstream of the *bla*_OXA-23_ gene (10), the other three possess IS*Aba1*. In Tn*2006*, the two IS*Aba1* copies are inversely oriented, contrasting with the same-direction orientation found in Tn*2009* (11).

The *bla*_OXA-23_ gene was either located on the chromosome or on plasmids. In 2024, Wang et al. (12) systematically analyzed the genomic diversity of global CRAB strains harboring the chromosome-borne *bla*_OXA-23_ gene, providing a comprehensive view of its chromosomal distribution. In contrast, plasmid-borne *bla*_OXA-23_ has received considerably less systematic attention. Previous studies have primarily focused on individual plasmids or specific strains. For example, Mugnier et al. (13) identified a CRAB isolate carrying the plasmid-borne *bla*_OXA-23_ gene on a 70-kb transferable plasmid. Liu et al. (14) reported that one *bla*_OXA-23_-carrying conjugative plasmid pAZJ221 was found in 23 *A. baumannii* and 3 *Acinetobacter nosocomialis* isolates. Xu et al. (15) reported a putative conjugative *bla*_OXA-23_-carrying plasmid pSRM1.1 in a CRAB strain SRM1. Although these case studies have provided valuable insights, a systematic, large-scale analysis of plasmid-borne *bla*_OXA-23_ is still lacking.

Resistance plasmids, particularly conjugative types, are primary drivers in the dissemination of multidrug resistance (MDR), as they often carry and co-transfer multiple resistance genes between bacteria (16). The conjugative transfer region of conjugative plasmids typically comprises four modules: origin of transfer (*oriT*), relaxase gene, type IV coupling protein (T4CP) gene, and type IV secretion system (T4SS) gene cluster (17). However, a systematic understanding of conjugative *bla*_OXA-23_-carrying plasmids and their transfer modules is still lacking. However, a systematic understanding of the conjugation modules and transfer capabilities of *bla*_OXA-23_-carrying plasmids remains absent.

In this study, we systematically analyzed the genomic characteristics of the *bla*_OXA-23_-carrying plasmids with complete sequences retrieved from the RefSeq database.

## RESULTS

### General characteristics of *bla*_OXA-23_-carrying plasmids available in the National Center for Biotechnology Information (NCBI) RefSeq database

Using ABRicate, 101 *bla*_OXA-23_-carrying plasmids with complete sequences were identified from the NCBI RefSeq database.

**Fig 1:**
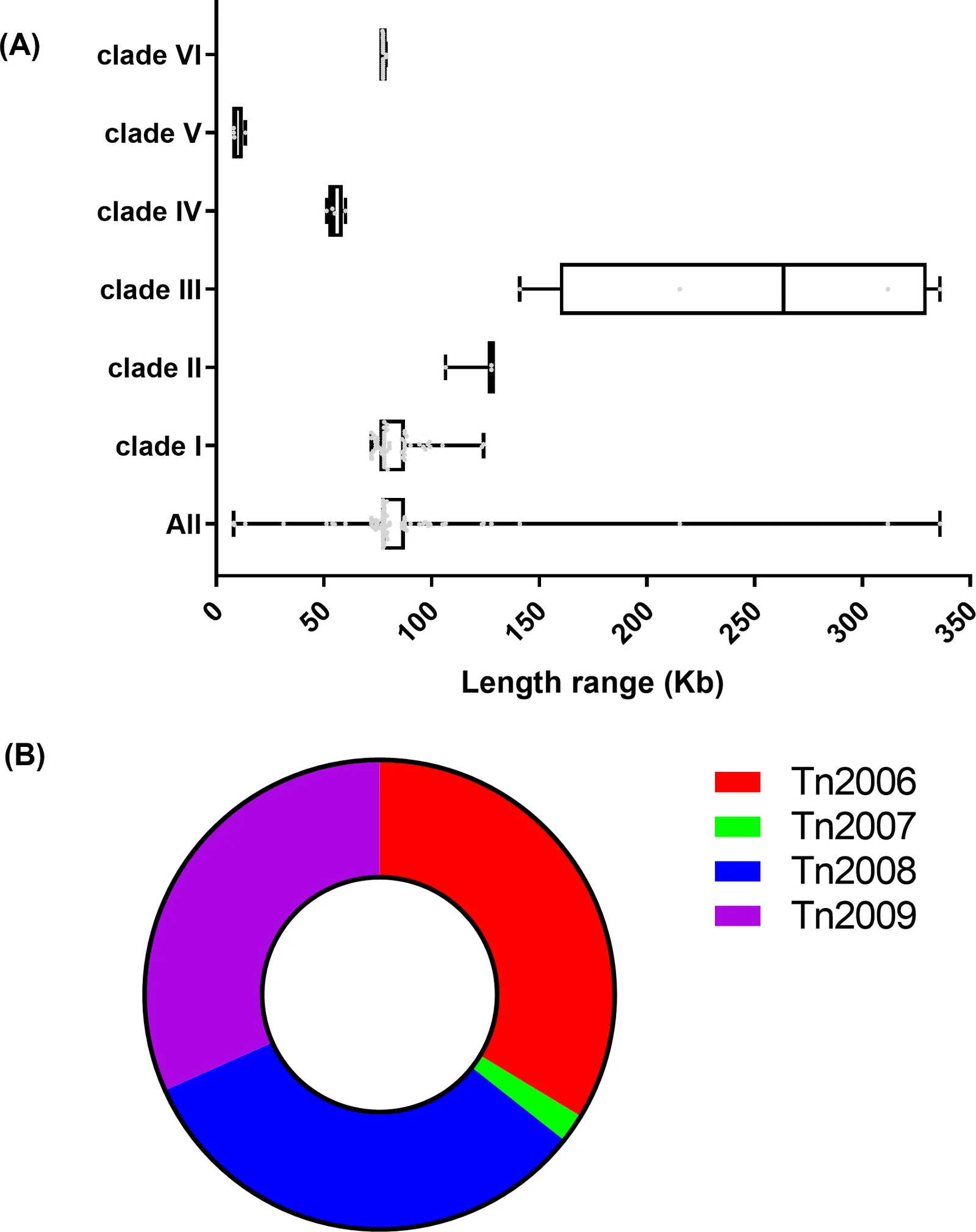
Characteristics of the 101 *bla*_OXA-23_-carrying plasmids retrieved from the NCBI RefSeq database. (**A**) Box plot showing the size distribution of the 101 *bla*_OXA-23_-carrying plasmids overall and across clades I–VI. (**B**) Pie chart showing the proportions of Tn*2006*, Tn*2007*, Tn*2008*, and Tn*2009* among the plasmids.

By analyzing the taxonomic distribution of bacterial strains harboring *bla*_OXA-23_-carrying plasmids, we observed that the 101 plasmids were present in seven different species (Fig. 1). The predominant host of these plasmids was *Acinetobacter baumannii*, which accounted for 94.06% (95 plasmids). The *bla*_OXA-23_-carrying plasmids were also identified in six other species, namely *Acinetobacter johnsonii*, *Acinetobacter pittii*, *Acinetobacter wuhouensis*, *Acinetobacter seifertii*, *Acinetobacter* sp. SCLZS86, and *P. mirabilis*.

We characterized the sizes of 101 complete-sequence plasmids carrying the *bla*_OXA-23_ gene. Their lengths ranged from 8.22 to 336.05 kb, with the 25th percentile, median, and 75th percentile being 76.52, 77.94, and 87.51 kb, respectively (Fig. 1A).

Based on the PlasmidFinder analysis, no plasmid replicon was identified in the 101 *bla*_OXA-23_-carrying plasmids, likely due to the database bias of PlasmidFinder toward Enterobacterales replicon types, which limits its effectiveness for *Acinetobacter* plasmids; however, replicon types were successfully determined for 91 of these plasmids using BLASTn against the AcinetobacterPlasmidTyping database. Among these, 85 plasmids belonged to the RP Rep family, and 6 belonged to the R3 Rep family. The 85 RP-family plasmids comprised 62 of the RP-T1 type and 23 of the RP-T2 type. These RP-family plasmids are characterized by replication initiation genes encoding Rep proteins belonging to the RepPriCT_1 family (Pfam: PF03090). A critical finding of the replicon typing analysis was the assignment of these two major RP-family groups to specific plasmid taxonomic units (PTUs): all 62 RP-T1 plasmids were assigned to PTU-Pse5, and all 23 RP-T2 plasmids were assigned to PTU-Pse1. Among the six R3-family plasmids, one was R3-T1, three were R3-T3, one was R3-T4, and one was R3-T33.

Notably, the *bla*_OXA-23_ gene was identified within composite transposons—specifically Tn*2006*, Tn*2007*, Tn*2008*, or Tn*2009*—in every one of the 101 plasmids examined. Among these genetic contexts, Tn*2006* (33.67%), Tn*2008* (32.67%), and Tn*2009* (31.68%) were predominant, while Tn*2007* was rare (1.98%) (Fig. 1B).

### Genomic diversity of the *bla*_OXA-23_-carrying plasmids

**Fig 2:**
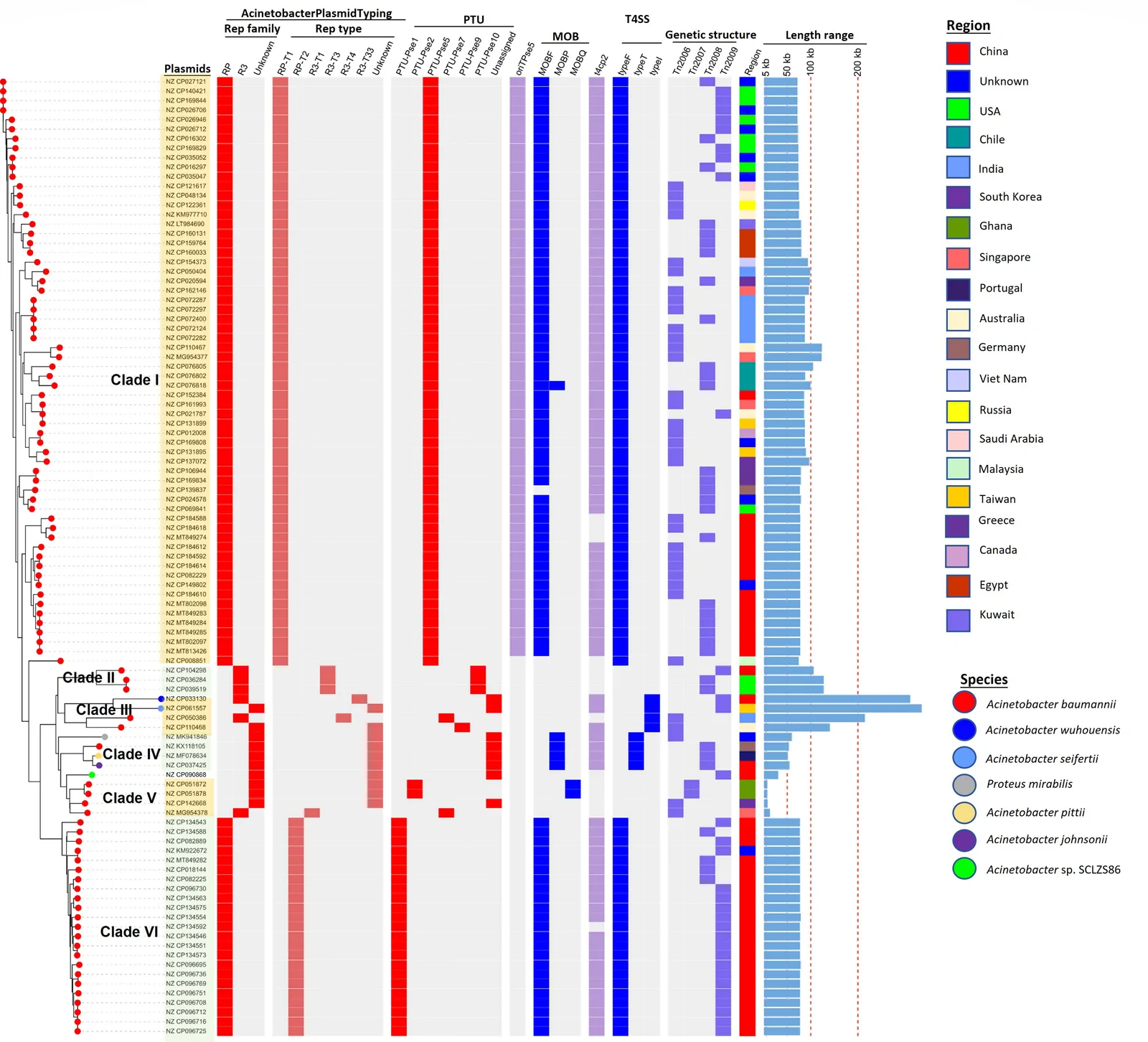
Gene-content-based dendrogram and associated features of the 101 *bla*_OXA-23_-carrying plasmids. The dendrogram was constructed based on the presence/absence of orthologous gene families. Surrounding tracks display replicon types, PTU assignments, predicted conjugation modules (*oriT*, relaxase, T4CP, and T4SS), transposon contexts (Tn*2006*, Tn*2007*, Tn*2008*, and Tn*2009*), geographical origins, and plasmid sizes. Colored dots on the dendrogram indicate host species (see legend on the right).

We constructed a gene-content-based dendrogram of the 101 *bla*_OXA- 23_-carrying plasmids to obtain a comprehensive overview of the 101 *bla*_OXA-23_-carrying plasmid genes. Based on the gene-content-based dendrogram combined with the information about the replicon types, lengths, genetic contexts of *bla*_OXA-23_, conjugative regions, as well as the geographical locations, most of the 101 *bla*_OXA-23_-carrying plasmids were clustered into six main clades (clades I–VI) (Fig. 2), representing six plasmid patterns carrying *bla*_OXA-23_ in *A. baumannii* and six other species. A concise summary of the core features of all six clades is provided in Table 1.

**TABLE 1:** Summary of the *bla*_OXA-23_-carrying plasmids

Clade	Plasmid numbers	Rep type	PTU	Plasmid sizes (kb)	Main geographic distribution	Transposon types	Conjugative transfer region			
							oriT	Relaxase	T4CP	T4SS
I	62	RP-T1	PTU-Pse5	72.11–124.11	China, USA, Australia, India, and Singapore	Tn*2006*, Tn*2008*, and Tn*2009*	*oriT*_Pse5_	MOB_F_	t4cp2	type F
II	3	R3-T3	PTU-Pse10	106.59–127.78	USA and China	Tn*2008* and Tn*2009*	–^*a*^	–	–	–
III	4	R3-T33, R3-T4, Unknown	PTU-Pse9, PTU-Pse7, Unassigned	86.14–130.72	Australia, India, and China	Tn*2006* and Tn*2009*	–	–	t4cp2	Type I
IV	4	–	–	51.29–60.02	USA and China	Tn*2006* and Tn*2008*	–	MOB_P_	t4cp2	Type T
V	4	R3-T1, Unknown	PTU-Pse2, PTU-Pse7, Unassigned	8.22–13.55	Ghana, South Korea, and Singapore	Tn*2006* and Tn*2007*	–	MOB_Q_	–	–
VI	23	RP-T2	PTU-Pse1	77.21–78.94	Mainly in China	Tn*2009* and Tn*2008*	–	MOB_F_	t4cp2	Type F

^*a*^ "–” indicates no corresponding result was obtained from the analysis.

### Clade I: *bla*_OXA-23_-carrying plasmids of RP-T1 type in *A. baumannii*

**Fig 3:**
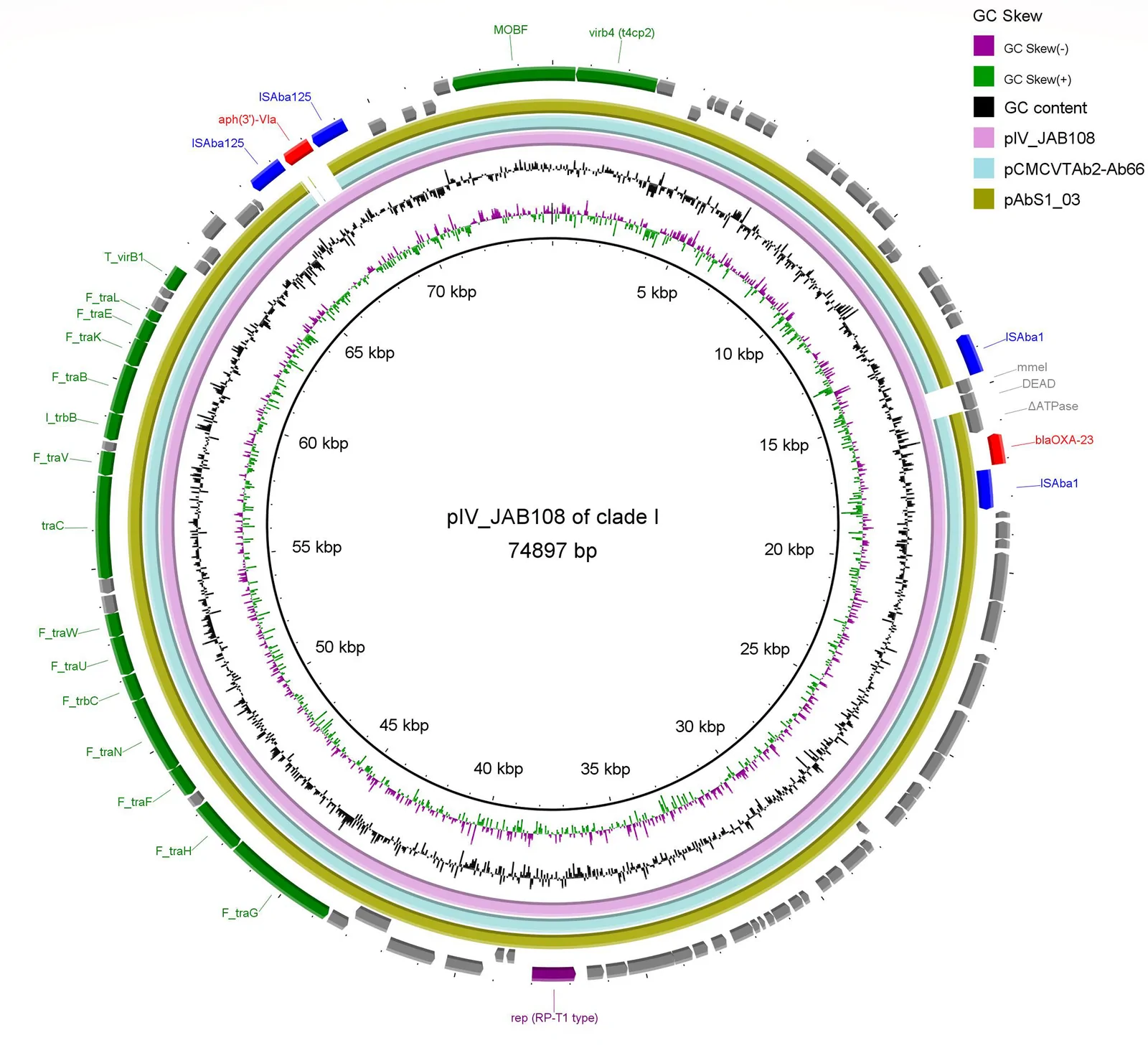
Comparative analysis of the conjugative transfer regions in representative clade I plasmids. The figure shows a BRIG comparison of three clade I plasmids carrying different transposon types. pIV_JAB108 (NZ_CP121617) was used as the reference plasmid; pCMCVTAb2-Ab66 (NZ_CP016302) and pAbS1_03 (NZ_CP026946) were the query plasmids. The *bla*_OXA-23_ gene was situated on Tn*2006*, Tn*2008*, and Tn*2009*, respectively. Resistance, transposase, and conjugative transfer genes were shown in red, blue, and green arrows, respectively.

A total of 62 *bla*_OXA-23_-carrying plasmids of RP-T1 type (PTU-Pse5) were identified in clade I, all hosted by *A. baumannii* (Fig. 2). For the 62 plasmids carrying *bla*_OXA-23_, their genome sizes varied from 72.11 to 124.11 kb (25th percentile = 75.81 kb; median = 78.03 kb; and 75th percentile = 87.53 kb) (Fig. 1 and 2). For the predicted conjugation modules, *oriT*_Pse5_ was identified in 61 of the 62 clade I plasmids, with one exception. In addition, almost all clade I plasmids carried genes encoding relaxases of the MOB_F_ family characterized by the domain “TrwC (Pfam: PF08751)” and T4CPs of t4cp2 characterized by the domain “TrwB_AAD_bind (PF10412)” (Fig. 2 and 3). Notably, all 62 clade I plasmids carried type F T4SS gene clusters (Fig. 2 and 3). The presence of *oriT*_Pse5_, together with genes encoding a relaxase, a T4CP, and a T4SS, strongly supports the classification of these plasmids as putatively conjugative. The plasmids in clade I were detected in several countries represented in the database, including China, the USA, Australia, India, and Singapore. For the genetic environment surrounding *bla*_OXA-23_ carried by the 62 plasmids of clade I in *A. baumannii*, *bla*_OXA-23_ was mainly located on the composite transposon Tn*2006* (29 plasmids) and Tn*2008* (24 plasmids) (Fig. 2), with the remaining 9 plasmids carrying Tn*2009* (Fig. 2).

### Clade II: *bla*_OXA-23_-carrying plasmids of R3-T3 type in *A. baumannii*

Three *bla*_OXA-23_-carrying plasmids of R3-T3 type (PTU-Pse10) with lengths ranging from 106.59 to 127.78 kb were classified into a small cluster, clade II of the gene-content-based dendrogram constructed with the 101 *bla*_OXA-23_-carrying plasmids, all found in *A. baumannii* strains (Fig. 2). None of the three plasmids of clade II were found to carry genes associated with conjugation or *oriT*. Two of the *bla*_OXA-23_-carrying plasmids of clade II were found in the USA and one found in China. For the two plasmids of clade II found in the USA, their *bla*_OXA-23_ were situated on the Tn*2008* (Fig. 2). For the plasmid of clade II found in China, the *bla*_OXA-23_ was situated on the Tn*2009* (Fig. 2).

### Clade III: mega *bla*_OXA-23_-carrying plasmids with type I T4SS genes

**Fig 4:**
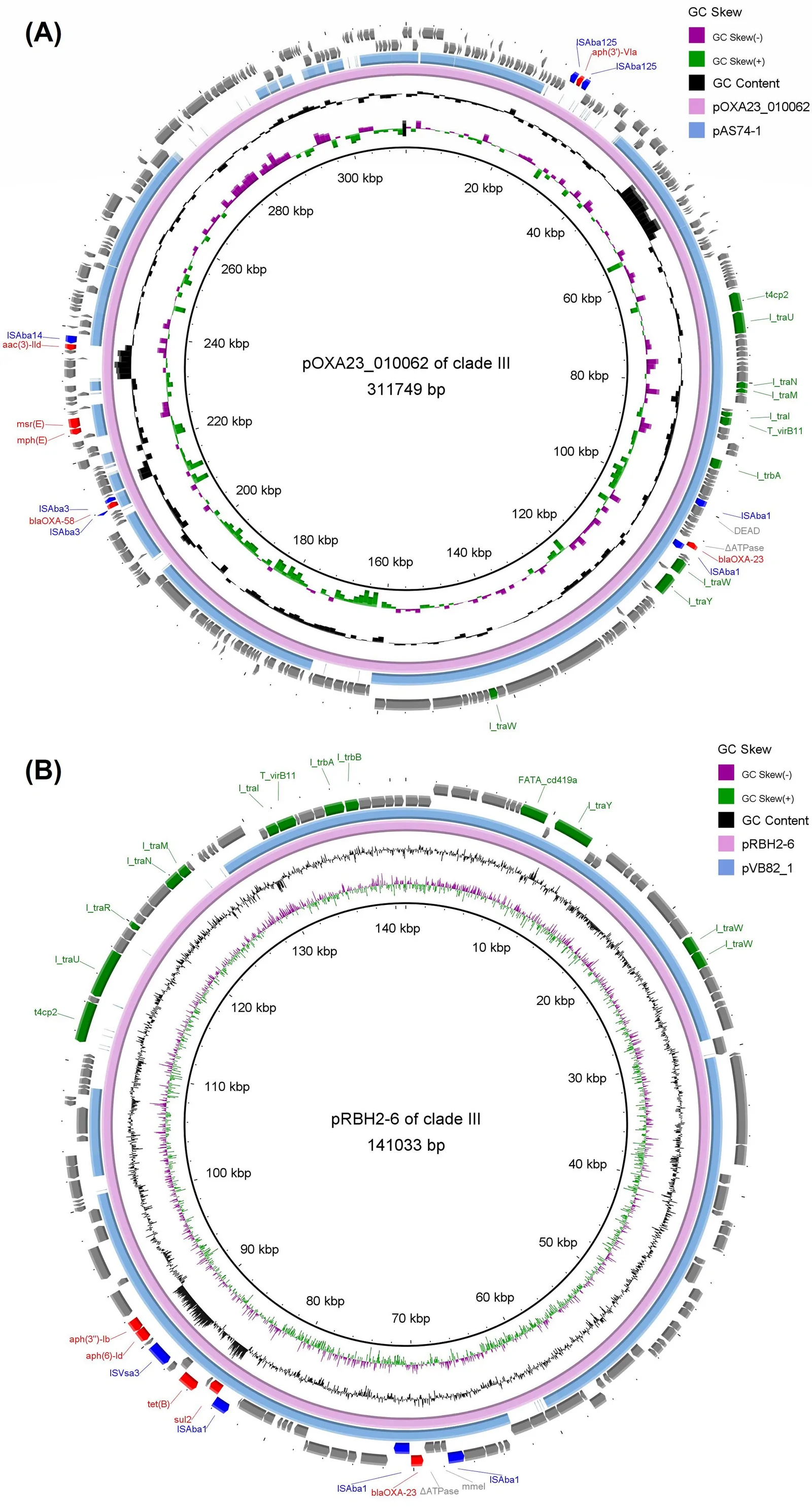
Comparative analysis of the conjugative transfer regions in plasmids of clade III. (**A**) Comparison of two plasmids of clade III harboring Tn*2009*. (**B**) Comparison of two plasmids of clade III harboring Tn*2006*. Resistance, transposase, and conjugative transfer genes were shown in red, blue, and green arrows, respectively.

One cluster of the gene-content-based dendrogram (clade III) composed of four mega *bla*_OXA-23_-carrying plasmids, including one 141.03-kb plasmid from *A. baumannii* found in Australia, one 215.28-kb plasmid from *A. baumannii* found in India, one 311.75-kb plasmid from *A. wuhouensis* found in Sichuan of China, and one 336.05-kb plasmid from *A. seifertii* found in Taiwan of China (Fig. 2). All of the plasmids of clade III were found to carry genes encoding type I T4SS, and three of the four plasmids of clade III carried genes encoding T4CPs of t4cp2 (Fig. 2 and 4). For the two plasmids of clade III found in China, their *bla*_OXA-23_ were situated on the Tn*2009* (Fig. 2 and 4), and the *bla*_OXA-23_ of the other two plasmids were situated on the Tn*2006* (Fig. 2 and 4).

### Clade IV: *bla*_OXA-23_-carrying plasmids with type T T4SS genes

**Fig 5:**
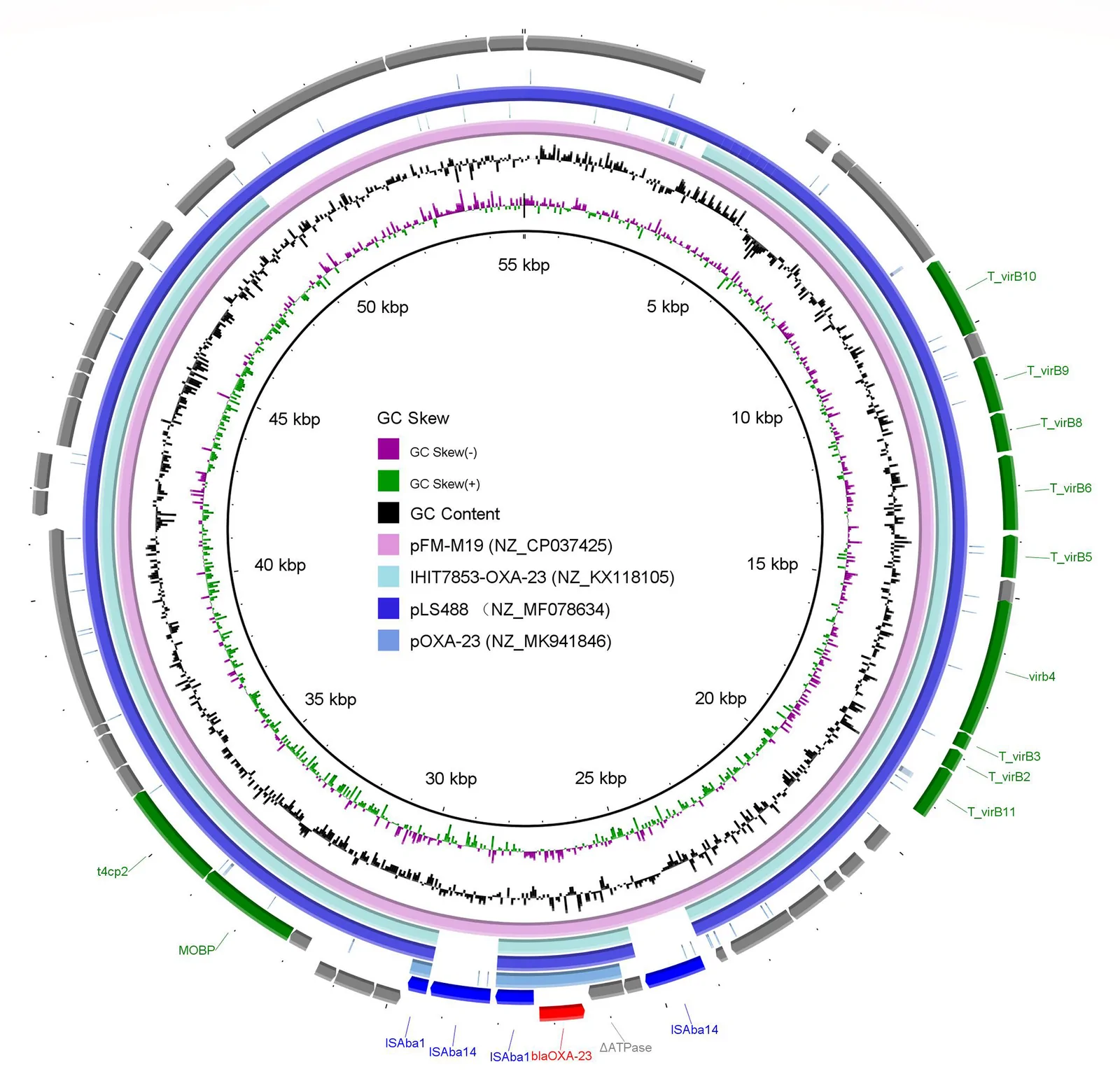
Comparative analysis of the conjugative transfer regions in clade IV plasmids. The figure shows a comparison of pFM-M19 (NZ_CP037425) with three other clade IV plasmids. Genes were color-coded as follows: resistance genes (red), transposase genes (blue), and conjugative transfer genes (green).

Four 50–60 kb *bla*_OXA-23_-carrying untypeable plasmids were classified into a small cluster, clade IV, found in four different species (*P. mirabilis*, *A. baumannii*, *A. johnsonii*, and *A.pittii*) (Fig. 2). All of the four plasmids of clade IV were found to carry genes encoding relaxases of MOB_P_ family characterized by the domain “Relaxase (Pfam: PF03432),” T4CPs of t4cp2, and type T T4SS (Fig. 2 and 5), inferred as putatively conjugative plasmids. Two of the *bla*_OXA-23_-carrying plasmids of clade IV were found in the USA and one found in China. For the genetic environment surrounding *bla*_OXA-23_ carried by the four plasmids of clade IV, the *bla*_OXA-23_ of three plasmids were located on the composite transposon Tn*2008*, and one plasmid carrying Tn*2006* (Fig. 2).

### Clade V: small *bla*_OXA-23_-carrying plasmids in *A. baumannii*

Four small plasmids with lengths ranging from 8.22 to 13.55 kb were grouped into the clade V of the gene-content-based dendrogram, all hosted by *A. baumannii* (Fig. 2), including two 8.22-kb plasmids found in Ghana, one 8.46-kb plasmid found in South Korea, and one 13.55-kb plasmid found in Singapore. For the two plasmids of clade V found in Ghana, their *bla*_OXA-23_ were situated on the Tn*2007* (Fig. 2). For the other plasmids of clade V found in China, the *bla*_OXA-23_ was situated on the Tn*2006* (Fig. 2).

### Clade VI: plasmids of RP-T2 type carrying *bla*_OXA-23_ in *A. baumannii*

**Fig 6:**
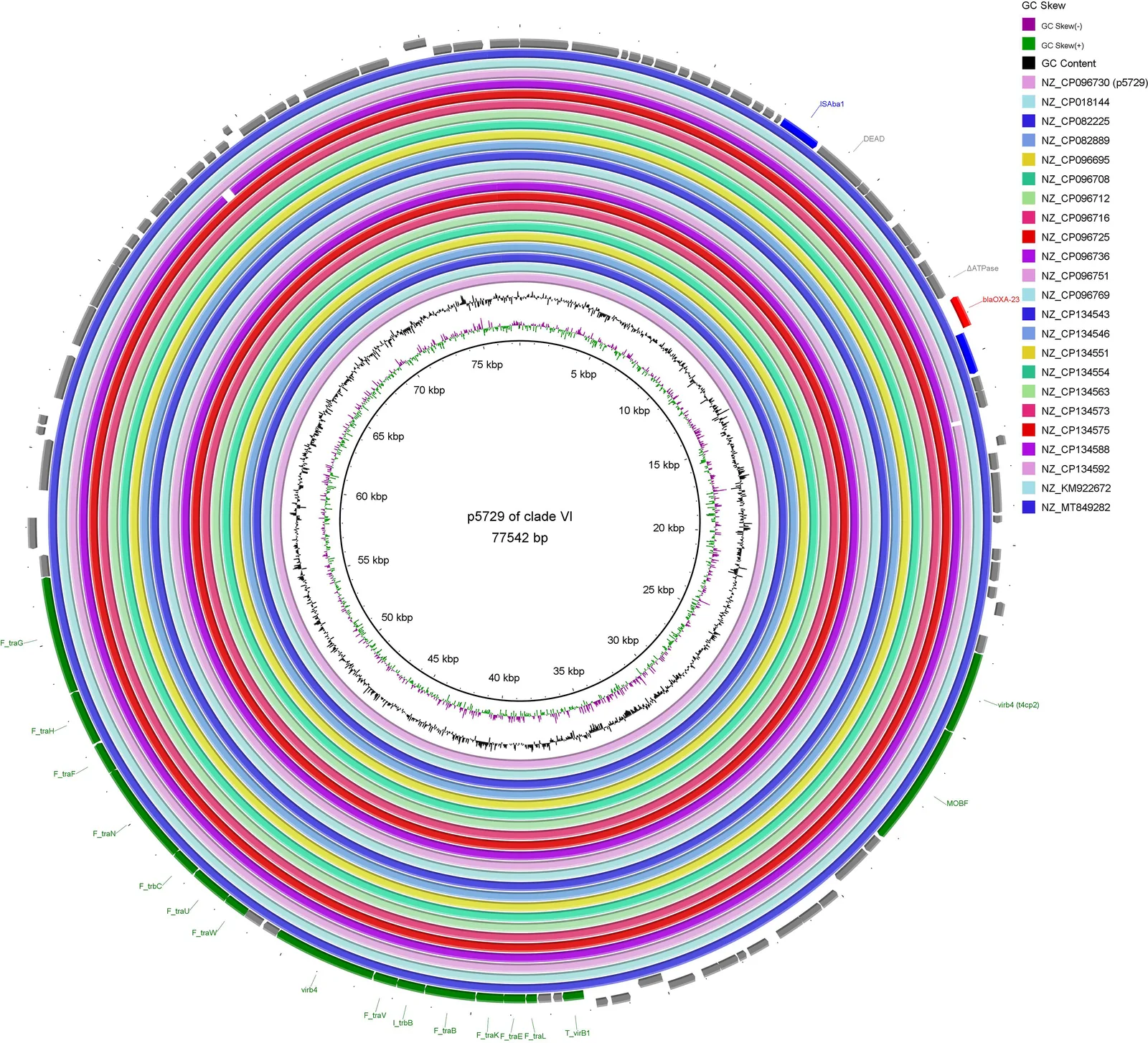
Comparative analysis of the conjugative transfer regions in clade VI plasmids. The figure shows a comparison of p5729 (NZ_CP096730) with 22 other clade VI plasmids. Genes are color-coded as follows: resistance genes (red), transposase genes (blue), and conjugative transfer genes (green).

Clade VI comprised 23 *bla*_OXA-23_-carrying plasmids of RP-T2 type (PTU-Pse1), all hosted by *A. baumannii* (Table 1). These plasmids exhibited a narrow size range of 77.21–78.94 kb (Fig. 1A). For the conjugative transfer regions, almost all the clade VI plasmids carried genes encoding relaxases of the MOB_F_ family, T4CPs of t4cp2, and type F T4SS gene clusters (Fig. 2 and 6). Although no definitive *oriT* site was identified on the clade VI plasmids, the presence of genes encoding a relaxase, a type T4CP, and a T4SS in almost all of them suggests that these plasmids are putatively conjugative. The plasmids of clade VI were mainly discovered in China, suggesting a more regionally restricted dissemination pattern. For the genetic context of *bla*_OXA-23_, the majority (19/23, 82.61%) carried Tn*2009*, with 19 plasmids carried Tn*2009*, while the remaining 4 plasmids (17.39%) harbored Tn*2008* (Fig. 2).

## DISCUSSION

In this study, we analyzed the genomic characteristics and diversity of 101 *bla*_OXA-23_-carrying plasmids with complete sequences retrieved from the RefSeq database. Notably, the vast majority (94.06%) of *bla*_OXA-23_-carrying plasmids were harbored by *A. baumannii*, underscoring that they are a key factor in the formation of CRAB. *A. baumannii* is an important cause of nosocomial infections worldwide (18), particularly among critically ill patients in intensive care units (19). Analyzing CRAB isolates over 65 months from a Beijing hospital, Ning et al. (20) found that the plasmid-borne *bla*_OXA-23_ gene was confined to specific sequence types (ST191, ST218, and ST368) of *A. baumannii*. Besides *A. baumannii*, the *bla*_OXA-23_-carrying plasmids were also detected in six other species, comprising five additional *Acinetobacter* species and *P. mirabilis*. This highlights the need for enhanced surveillance of *bla*_OXA-23_-carrying plasmids to mitigate the risk of their cross-species transmission, especially the conjugative plasmids with high conjugation ability (14).

Cluster analysis of 101 *bla*_OXA-23_-carrying plasmids identified two major plasmid types: 62 belonged to the RP-T1 (PTU-Pse5) type, and 23 to the RP-T2 (PTU-Pse1) type. The RP-T1 type exhibited a wider geographical distribution in the current data set, while dissemination of the RP-T2 type was primarily confined to China. However, given the inherent sampling bias of the RefSeq database, these observations should be interpreted with caution and confirmed by future studies with broader sampling coverage. The RP-T1 plasmids, typified by pAb-G7-2 (21), harbor conjugation genes and are frequently associated with diverse acquired antibiotic resistance genes (22). RP-T1 plasmids carrying *bla*_OXA-23_, with genome sizes ranging from 72.11 to 124.11 kb, possess a conserved core structure (e.g., *rep* gene and conjugation regions), implying a history of evolution through processes like gene loss or acquisition (23). The *bla*_OXA-23_-carrying RP-T2 plasmids endemic to China are primarily represented by a single ~78 kb conjugative plasmid, pAZJ221, which was characterized in an investigation of CRAB from 28 hospitals in 18 provinces of China in 2015 (14). Notably, both the RP-T1 and the RP-T2 plasmids carrying *bla*_OXA-23_, which were classified as putatively conjugative, shared the same predicted conjugation modules, including genes encoding relaxases of the MOB_F_ family, T4CPs of t4cp2, and type F T4SS. The MOB_F_ family, which was characterized by the domain “TrwC (PF08751),” with R388 TrwC and F TraI as representatives (24). The T4CPs of t4cp2 were characterized by the domain “TrwB_AAD_bind (PF10412),” with the TrwB encoded by plasmid R388 as a representative (25). The type F T4SS is typified by that of the F plasmid, whose T4SS is encoded by 18 core genes (26).

In addition to the two dominant lineages, clade IV comprised four 50–60 kb untypeable *bla*_OXA-23_-carrying plasmids spanning four distinct species (*P. mirabilis*, *A. baumannii*, *A. johnsonii*, and *A. pittii*). These plasmids carried MOB_P_ relaxases, t4cp2-type T4CPs, and type T T4SS, inferred as putatively conjugative in this study. Notably, plasmid pFM-M19, a representative of this clade isolated from *A. johnsonii*, has been experimentally validated as fully conjugative, transferring *bla*_OXA-23_ to *E. coli* strain 25DN via conjugation and producing carbapenem-resistant transconjugants in classic mating assays (27). This experimental validation reinforces our bioinformatic predictions regarding the mobility of clade IV plasmids.

The *bla*_OXA-23_ gene, originally an intrinsic chromosomal gene of *Acinetobacter radioresistens* expressed at low levels (28), was mobilized into pathogenic *Acinetobacter* species and other gram-negative hosts primarily via IS*Aba1*-flanked composite transposons. In this study, we found that the plasmid-borne *bla*_OXA-23_ gene was mainly located within Tn*2006* (33.67%), Tn*2008* (32.67%), and Tn*2009* (31.68%), while only a small subset was associated with Tn*2007*. In Tn*2006*, Tn*2008*, and Tn*2009*, the plasmid-borne *bla*_OXA-23_ gene was invariably preceded by an upstream IS*Aba1* element. IS*Aba1*, an IS*4* family insertion sequence characterized by 16-bp imperfect inverted repeats and generating 9-bp target site duplications (29), not only acts as a structural boundary for transposition but also carries highly active promoter sequences that drive high-level overexpression of the carbapenemase when inserted upstream of *bla*_OXA-23_ (30). In Tn*2007*, however, *bla*_OXA-23_ is associated with an upstream IS*Aba4* insertion (10). Belonging to the IS*982* family, this 975-bp IS*Aba4* carries 18-bp IRs and encodes a 292-amino-acid transposase, but generates no target site duplication and lacks the strong promoter properties of IS*Aba1* (29). The marked predominance of IS*Aba1*-associated transposons in our data set is therefore likely explained by this functional distinction: IS*Aba1*’s ability to upregulate resistance gene expression upon insertion confers a significant selective advantage to carbapenem-resistant isolates, whereas IS*Aba4* does not provide such a benefit.

## MATERIALS AND METHODS

### Retrieval of bacterial plasmid sequences from the NCBI RefSeq database

Bacterial plasmid genomic sequences without duplicates were downloaded from the National Center for Biotechnology Information (NCBI) RefSeq database (https://ftp.ncbi.nih.gov/refseq/release/plasmid/), and the download date was 10 June 2025.

### Identification of *bla*_OXA-23_-carrying plasmids in the NCBI RefSeq database

The *bla*_OXA-23_ gene carried by the plasmids was identified using ABRicate (https://github.com/tseemann/abricate) with 100.00% identity and 100.00% coverage. The plasmids carrying *bla*_OXA-23_ with a complete sequence were selected for further study.

### Bioinformatic analyses of the *bla*_OXA-23_-carrying plasmids

Plasmid replicon typing of the *bla*_OXA-23_-carrying plasmids was performed using BLASTn against the PlasmidFinder (31) and AcinetobacterPlasmidTyping database (22), respectively. The *bla*_OXA-23_-carrying plasmids were further classified into plasmid taxonomic units (PTUs) using software COPLA (32). Insertion sequences (ISs) adjacent to the *bla*_OXA-23_ gene in the genomes of the *bla*_OXA-23_-carrying plasmids were identified using BLASTn against the ISfinder (33) database. The transposon types (Tn*2006–Tn2009*) were assigned based on the structural criteria described by Nigro and Hall (28). The potential conjugative transfer region (*oriT*, relaxase gene, T4CP gene, and T4SS gene cluster) of *bla*_OXA-23_-carrying plasmids was analyzed using the software oriTfinder2 (34). As the currently available oriTfinder2 database does not yet contain the recently characterized *oriT*_Pse5_ sequence (35), we manually added this reference sequence to our local database prior to performing a targeted BLASTn search against all plasmid sequences. The comparison of plasmids was conducted and visualized using Easyfig version 2.2.5 (36) and BRIG version 0.95 (37). For the gene-content-based dendrogram of the *bla*_OXA-23_-carrying plasmids, a binary matrix of orthologous gene family presence/absence was constructed with OrthoFinder (38), followed by hierarchical clustering via PAST5 (39) and visualization of the resulting gene-content-based dendrogram using iTOL (40).

Information on geographic location, host organism, and plasmid size for the *bla*_OXA-23_-carrying plasmids was primarily extracted from the genomic annotations in NCBI GenBank files. In instances where records were incomplete for certain metrics, the relevant information was sought by manually reviewing the original publications associated with each plasmid, as identified by the article titles provided in the GenBank records.

## Data Availability

All complete plasmid sequences carrying *bla*_OXA-23_ analyzed in this study were retrieved from the NCBI RefSeq database (https://www.ncbi.nlm.nih.gov/refseq/). A total of 101 RefSeq accession numbers were included in this analysis. The complete list of accession numbers is provided in Table S1.

## SUPPLEMENTAL MATERIAL

Table S1 spectrum.01374-26-s0001.docx List of 101 complete *bla*_OXA-23_-carrying plasmid sequences from the NCBI RefSeq database, with accession numbers, organism sources, strain names, and plasmid designations.

ASM does not own the copyrights to Supplemental Material that may be linked to, or accessed through, an article. The authors have granted ASM a non-exclusive, world-wide license to publish the Supplemental Material files. Please contact the corresponding author directly for reuse.
