# Supplementary material for: Comparative genomic analysis of the blaOXA-23-carrying plasmids
Source: Microbiol Spectr. 2026 Sep 29;14(11):e01374-26. doi: 10.1128/spectrum.01374-26 (PMC13729139; doi:10.1128/spectrum.01374-26)
Supplement: Supplementary file 1 [file spectrum.01374-26-s0001.docx]

**Comparative Genomic Analysis of the *bla*_OXA-23_-Carrying plasmids**

**Table S1**. List of 101 complete *bla*_OXA-23_-carrying plasmid sequences from the NCBI RefSeq database, with accession numbers, organism sources, strain names, and plasmid designations.

| Accession | *organism* | strain | plasmid |
| --- | --- | --- | --- |
| NZ_CP090868 | *Acinetobacter sp.* SCLZS86 | SCLZS86 | unnamed |
| NZ_CP152384 | *Acinetobacter baumannii* | SRM1 | pSRM1.1 |
| NZ_MK941846 | *Proteus mirabilis* | PM1157 | pOXA-23 |
| NZ_CP154373 | *Acinetobacter baumannii* | NCSR_106 | pNCSR_106-5 |
| NZ_KX118105 | *Acinetobacter baumannii* | IHIT7853 | IHIT7853-OXA-23 |
| NZ_CP082225 | *Acinetobacter baumannii* | 2014BJAB1 | p2014BJAB1 |
| NZ_CP082889 | *Acinetobacter baumannii* | 2014LNAB1 | p2014LNAB1-2 |
| NZ_CP096751 | *Acinetobacter baumannii* | 5666 | p5666_2 |
| NZ_CP096716 | *Acinetobacter baumannii* | 5745 | p5745_2 |
| NZ_CP096730 | *Acinetobacter baumannii* | 5729 | p5729 |
| NZ_CP096695 | *Acinetobacter baumannii* | 5773 | p5773_2 |
| NZ_CP096712 | *Acinetobacter baumannii* | 5759 | p5759_2 |
| NZ_CP096736 | *Acinetobacter baumannii* | 5683 | p5683_1 |
| NZ_CP096725 | *Acinetobacter baumannii* | 5734 | p5734_1 |
| NZ_CP096708 | *Acinetobacter baumannii* | 5760 | p5760_1 |
| NZ_CP096769 | *Acinetobacter baumannii* | 5388 | p5388_1 |
| NZ_CP121617 | *Acinetobacter baumannii* | JAB108 | pIV_JAB108 |
| NZ_CP082229 | *Acinetobacter baumannii* | 2018HLJAB1 | p2018HLJAB1 |
| NZ_CP159764 | *Acinetobacter baumannii* | AB11-AUFP | pAB11-AUFP1 |
| NZ_CP076805 | *Acinetobacter baumannii* | UC24371 | p1UC24371 |
| NZ_CP076818 | *Acinetobacter baumannii* | UC24137 | p1UC24137 |
| NZ_CP160033 | *Acinetobacter baumannii* | AB44-AUFP | pAB44-AUFP1 |
| NZ_CP122361 | *Acinetobacter baumannii* | GIMC5511:ABT-AB4 | pABT-AB4-1 |
| NZ_CP161993 | *Acinetobacter baumannii* | SIMBA034 | pSIMBA034_1 |
| NZ_CP076802 | *Acinetobacter baumannii* | UC25604 | p1UC25604 |
| NZ_CP162146 | *Acinetobacter baumannii* | SIMBA089 | pSIMBA089_1 |
| NZ_CP008851 | *Acinetobacter baumannii* | AC29 | pAC29b |
| NZ_CP061557 | *Acinetobacter seifertii* | AS74 | pAS74-1 |
| NZ_CP160131 | *Acinetobacter baumannii* | AB37-AUFP | pAB37-AUFP1 |
| NZ_CP110467 | *Acinetobacter baumannii* | RBH2 | pRBH2-5 |
| NZ_CP110468 | *Acinetobacter baumannii* | RBH2 | pRBH2-6 |
| NZ_CP169829 | *Acinetobacter baumannii* | Lv647 | unnamed1 |
| NZ_CP169834 | *Acinetobacter baumannii* | Rp376 | unnamed1 |
| NZ_CP169844 | *Acinetobacter baumannii* | Rp428 | unnamed2 |
| NZ_CP169808 | *Acinetobacter baumannii* | HvAb04 | unnamed3 |
| NZ_CP134551 | *Acinetobacter baumannii* | XH1048 | pXH1048 |
| NZ_CP134554 | *Acinetobacter baumannii* | XH1045 | pXH1045 |
| NZ_CP134592 | *Acinetobacter baumannii* | XH1019 | pXH1019_1 |
| NZ_CP134573 | *Acinetobacter baumannii* | XH1036 | pXH1036_2 |
| NZ_CP134575 | *Acinetobacter baumannii* | XH1035 | pXH1035_1 |
| NZ_CP134543 | *Acinetobacter baumannii* | XH1055 | pXH1055_3 |
| NZ_CP134563 | *Acinetobacter baumannii* | XH1040 | pXH1040 |
| NZ_CP134546 | *Acinetobacter baumannii* | XH1054 | pXH1054_2 |
| NZ_CP134588 | *Acinetobacter baumannii* | XH1026 | pXH1026_2 |
| NZ_CP106944 | *Acinetobacter baumannii* | ARC6851 | pARC6851_1 |
| NZ_CP142668 | *Acinetobacter baumannii* | C20AB12 | pC20AB12 |
| NZ_CP137072 | *Acinetobacter baumannii* | SNUBHAB0220 | pA0220 |
| NZ_CP069841 | *Acinetobacter baumannii* | FDAARGOS_1360 | unnamed1 |
| NZ_CP012008 | *Acinetobacter baumannii* | Ab04-mff | pAB04-2 |
| NZ_CP027121 | *Acinetobacter baumannii* | AR_0056 | unnamed2 |
| NZ_CP139837 | *Acinetobacter baumannii* | B84 | pB84_1 |
| NZ_CP140421 | *Acinetobacter baumannii* | 2023CK-00893 | unnamed1 |
| NZ_CP020594 | *Acinetobacter baumannii* | USA15 | pUSA15_1 |
| NZ_CP024578 | *Acinetobacter baumannii* | AbPK1 | pAbPK1b |
| NZ_CP036284 | *Acinetobacter baumannii* | TG60155 | p60155_1 |
| NZ_CP037425 | *Acinetobacter johnsonii* | M19 | pFM-M19 |
| NZ_CP033130 | *Acinetobacter wuhouensis* | WCHAW010062 | pOXA23_010062 |
| NZ_CP026712 | *Acinetobacter baumannii* | AR_0063 | unitig_2_pilon |
| NZ_CP026706 | *Acinetobacter baumannii* | AR_0056 | tig00000059_pilon |
| NZ_CP035052 | *Acinetobacter baumannii* | ABUH763 | p74.1Kbp |
| NZ_CP039519 | *Acinetobacter baumannii* | TG22653 | pTG22653 |
| NZ_CP035047 | *Acinetobacter baumannii* | ABUH793 | p74.1Kbp |
| NZ_CP026946 | *Acinetobacter baumannii* | S1, AB1A2 | pAbS1_03 |
| NZ_CP072124 | *Acinetobacter baumannii* | KSK1 | p2KSK1 |
| NZ_CP072400 | *Acinetobacter baumannii* | KSK2 | p2KSK2 |
| NZ_CP072297 | *Acinetobacter baumannii* | KSK19 | p2KSK19 |
| NZ_CP072287 | *Acinetobacter baumannii* | KSK11 | p2KSK11 |
| NZ_CP072282 | *Acinetobacter baumannii* | KSK10 | p2KSK10 |
| NZ_CP018144 | *Acinetobacter baumannii* | HRAB-85 | unnamed |
| NZ_CP051878 | *Acinetobacter baumannii* | Ab-B004d-c | pAb-B004d-c_3 |
| NZ_CP051872 | *Acinetobacter baumannii* | Ab-D10a-a | pAb-D10a-a_3 |
| NZ_CP050386 | *Acinetobacter baumannii* | VB82 | pVB82_1 |
| NZ_CP050404 | *Acinetobacter baumannii* | VB2486 | pVB2486_1 |
| NZ_KM922672 | *Acinetobacter baumannii* | A221 | pAZJ221 |
| NZ_KM977710 | *Acinetobacter baumannii* | D46 | pD46-3 |
| NZ_CP021787 | *Acinetobacter baumannii* | A85 | pA85-3 |
| NZ_CP048134 | *Acinetobacter baumannii* | D46 | pD46-3 |
| NZ_LT984690 | *Acinetobacter baumannii* | K50 | I |
| NZ_MF078634 | *Acinetobacter pittii* | HGSA488 | pLS488 |
| NZ_MT802097 | *Acinetobacter baumannii* | 5627 | unnamed |
| NZ_MT802098 | *Acinetobacter baumannii* | 5630 | unnamed |
| NZ_MT849274 | *Acinetobacter baumannii* | 2016BJAB1 | unnamed |
| NZ_MT849282 | *Acinetobacter baumannii* | 2014TJAB1 | unnamed |
| NZ_MT849283 | *Acinetobacter baumannii* | 2018TJAB1 | unnamed |
| NZ_MT849285 | *Acinetobacter baumannii* | 2018BJAB2 | unnamed |
| NZ_MT849284 | *Acinetobacter baumannii* | 2018BJAB1 | unnamed |
| NZ_CP016302 | *Acinetobacter baumannii* | CMC-CR-MDR-Ab66 | pCMCVTAb2-Ab66 |
| NZ_CP016297 | *Acinetobacter baumannii* | CMC-CR-MDR-Ab4 | pCMCVTAb2-Ab4 |
| NZ_MT813426 | *Acinetobacter baumannii* | 2018HBAB1 | p2 |
| NZ_CP184610 | *Acinetobacter baumannii* | CQPMC-AB12 | pCYPMC033144-137 |
| NZ_CP184588 | *Acinetobacter baumannii* | CQPMC-AB25 | pCYPMC033143-151 |
| NZ_CP184612 | *Acinetobacter baumannii* | CQPMC-AB11 | pCYPMC009739-108 |
| NZ_CP184592 | *Acinetobacter baumannii* | CQPMC-AB23 | pCYPMC125228-111 |
| NZ_CP184614 | *Acinetobacter baumannii* | CQPMC-AB10 | pCYPMC139061-141 |
| NZ_MG954377 | *Acinetobacter baumannii* | SGH9601 | pS21-2 |
| NZ_MG954378 | *Acinetobacter baumannii* | SGH0905 | pS32-1 |
| NZ_CP184618 | *Acinetobacter baumannii* | CQPMC-AB08 | pCYPMC033142-150 |
| NZ_CP149802 | *Acinetobacter baumannii* | AB7276 | poxa23 |
| NZ_CP104298 | *Acinetobacter baumannii* | ZHOU | pZHOU-1 |
| NZ_CP131895 | *Acinetobacter baumannii* | 2008C02-166 | p2008C02-166-2 |
| NZ_CP131899 | *Acinetobacter baumannii* | 2008N08-156 | p2008N08-156-1 |
